# Patent Foramen Ovale as a Rare Focus of Origination of Atrial Tachycardia

**DOI:** 10.19102/icrm.2019.100405

**Published:** 2019-04-15

**Authors:** Jian L. Tan, Sandeep Sharma

**Affiliations:** ^1^Department of Internal Medicine, Crozer-Chester Medical Center, Upland, PA, USA; ^2^Department of Cardiology, Crozer-Chester Medical Center, Upland, PA, USA

**Keywords:** Alternating cycle length, atrial tachycardia, catheter ablation, interatrial septum, patent foramen ovale

## Abstract

Supraventricular tachycardia refers to a group of arrhythmias whose mechanism involves tissues from the His bundle or above. Repetitive focal atrial tachycardia (AT) (FAT) accounts for less than 10% of supraventricular tachycardia cases. FAT originating from the patent foramen ovale (PFO) has not been well-described and is a rarely reported phenomenon to date. Here, we report a rare case of FAT arising from the PFO. To the best of our knowledge, this is the first detailed report of AT arising from the PFO. We have included the description of the ablation procedure and postulated the possible electrophysiological mechanisms of a regularly irregular FAT noted in our patient during the electrophysiology study. Our case shows catheter ablation to be a successful treatment strategy in AT arising from the PFO, with the possibility of providing a long-term cure and freedom from antiarrhythmic drugs.

## Introduction

Supraventricular tachycardia (SVT) is an umbrella term used to describe a group of arrhythmias whose mechanism involves tissue from the His bundle or above.^1^ The most common subtypes of SVT in descending order generally include atrial fibrillation, atrial flutter, atrioventricular nodal reentrant tachycardia (AVNRT), atrioventricular reentrant tachycardia, and atrial tachycardia (AT).^2^ Repetitive focal AT (FAT) is a relatively rare cause of SVT, accounting for less than 10% of SVT cases.^2,3^ FATs frequently originate from the right atrium versus the left atrium.^1,4^ Foci originating from the interatrial septum (IAS)^5,6^ and anterior atrial septum^7^ have been commonly reported, whereas foci originating from the peripatent foramen ovale (peri-PFO) have not been as well-described. Herein, we report a rare case of FAT originating from the peri-PFO and describe the possible electrophysiological mechanisms of a regularly irregular FAT.

## Case presentation

A 47-year-old morbidly obese woman with a significant history of diet-controlled type II diabetes mellitus and several episodes of unprovoked symptomatic palpitations since the age of 20 years presented with shortness of breath and recurrent palpitations to the emergency department. Two weeks before admission, she had experienced an episode of left lower anterior chest tightness that lasted for hours and was associated with shortness of breath and dizziness. Physical examination was significant for class III obesity, bibasilar crackles, S3 gallop, and bilateral trace pitting pedal edema.

**Figure 1:**
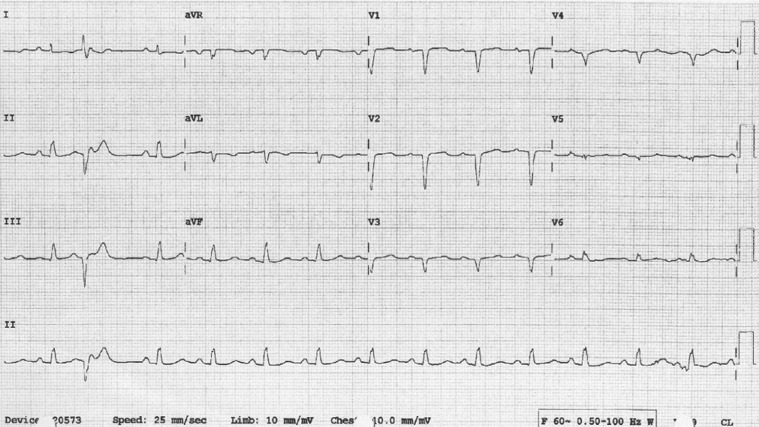
A 12-lead ECG during sinus rhythm at 83 bpm, low-amplitude QRS voltage, Q-waves in V1 through V4, and a premature ventricular complex.

**Figure 2:**
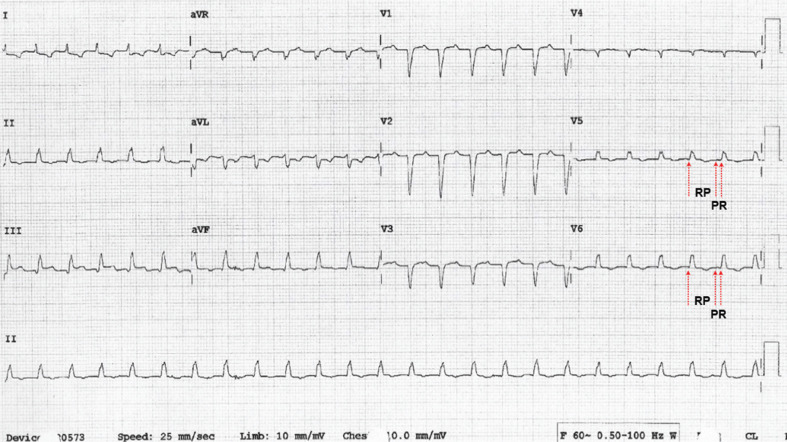
A 12-lead ECG during tachycardia (long-RP variant) at 146 bpm.

A 12-lead electrocardiogram (ECG) during sinus rhythm at a rate of 83 bpm was significant for pathological Q-waves in V1 through V4 suggestive of old anteroseptal myocardial infarction **(Figure 1)**. She developed tachycardia, with the 12-lead ECG showing long RP tachycardia at a rate of 146 bpm **(Figure 2)**. Her thyroid profile was normal, brain natriuretic peptide level was 369 pg/mL, and cardiac troponin was in the range of 0.06 ng/mL to 0.08 ng/mL.

Chest X-ray showed minimal bilateral pleural effusions and marked interstitial edema, consistent with congestive heart failure. Transthoracic echocardiogram (TTE) showed inferior wall akinesis with hypokinesis of the remaining segments, moderate left ventricular systolic dysfunction (ejection fraction: 35%–40%), and left atrium dilatation. The patient underwent cardiac catheterization that revealed diffuse multivessel coronary artery disease, and coronary artery bypass grafting (CABG) was planned.

She persistently developed recurrent symptomatic SVTs before surgery, which were all terminated with the administration of intravenous adenosine. The decision was made to perform a cardiac electrophysiology (EP) study and radiofrequency catheter ablation (RFCA) before the CABG procedure. For this, 8- and 7-French short sheaths were inserted into the right femoral vein and a 7-French short sheath was inserted into the left common femoral vein. An octapolar catheter was positioned in the coronary sinus (CS), a Josephson quadripolar catheter was positioned in the right ventricular apex, and a His catheter was positioned in the His-bundle region. Programmed electrical stimulation was performed, and tachycardia was induced with atrial burst pacing.

**Figure 3:**
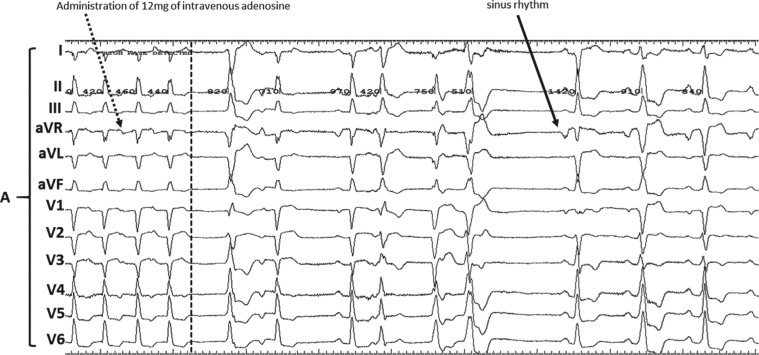
AT was successfully abrupted (dotted black line) with the administration of 12 mg of intravenous adenosine (dotted black arrow). “**A**” denotes the surface leads. Of note, the variability of the Q- and R-waves in lead V4 between **Figures 1** and **3** could be due to a difference in lead positioning and the patient’s body habitus.

**Figure 4:**
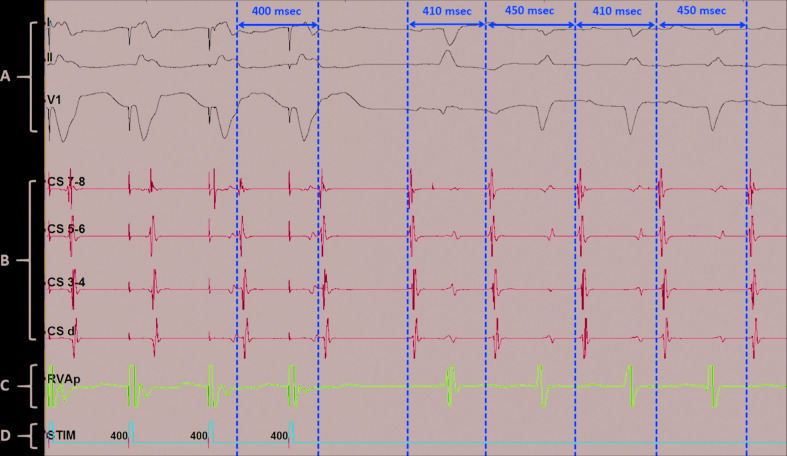
Electrogram was obtained with an octapolar catheter (Abbott Laboratories, Chicago, IL, USA) placed at the CS and a Josephson quadripolar catheter placed at the right ventricular apex, respectively. Right ventricular apical pacing at a cycle length of 400 ms induced a long-RP tachycardia, following an initial “V-A-A-V” electrogram sequence. The induced tachycardia had a bigeminal cycle length, vacillating every other beat at 410 ms followed by 450 ms. **A**: Surface leads; **B**: CS intracardiac leads; **C**: right ventricular intracardiac lead; **D**: programmed stimulation.

**Figure 5:**
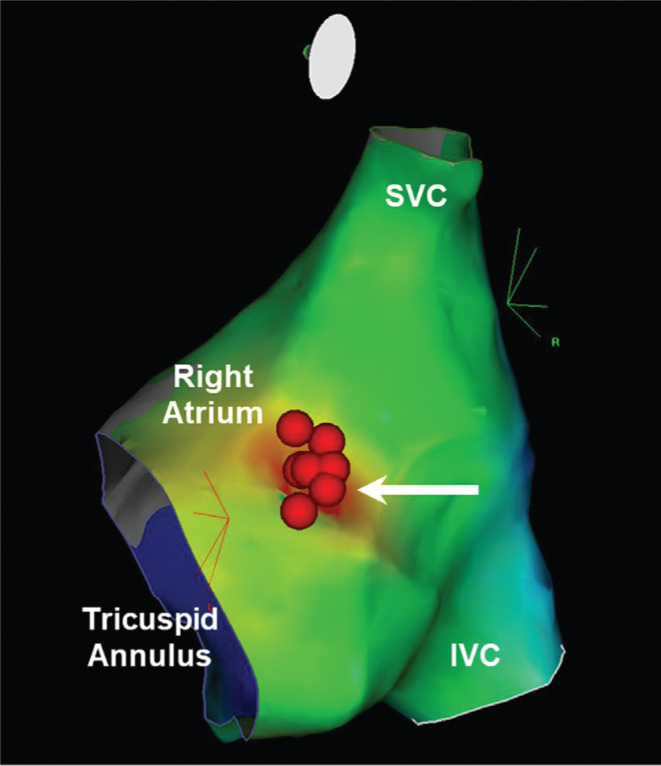
Right atrial activation map during FAT, right atrium view. Represented anatomic structures include the right atrium, superior vena cava, inferior vena cava, tricuspid orifice, and successful ablation of the AT at the peri-PFO (red dots). The earliest RA activation occurs over a focus area as marked by the red isochrone (white arrow) with emanation throughout the interatrial septum. SVC: superior vena cava; IVC: inferior vena cava.

**Figure 6:**
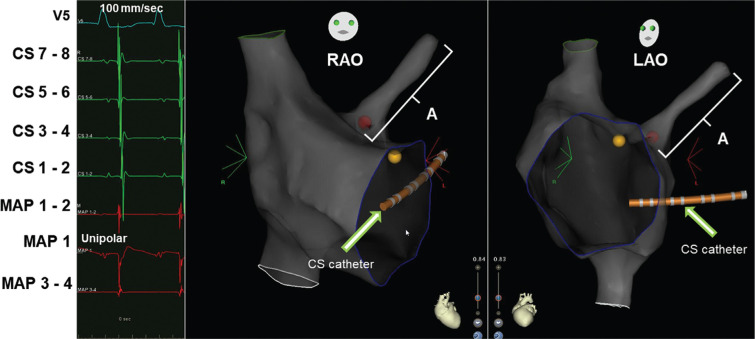
CARTO^®^ image (Biosense Webster, Diamond Bar, CA, USA) showing the successful ablation of the AT at the PFO (red dot), which was found to be the site of origin of AT. **A:** The catheter incidentally landed into the left atrium through the PFO and mapped part of the left atrium. Yellow dot: His cloud; red dot: terminated lesion at the peri-PFO; white brace: part of the mapped left atrium; RAO: right anterior oblique cranial projection; LAO: left anterior oblique cranial projection.

During tachycardia **(Figure 3)**, 12 mg of adenosine was administered intravenously, which resulted in abrupt termination of the tachycardia followed by ventricular and atrial ectopy and then resumption of sinus rhythm. The induced tachycardia had a cycle length (CL) of 410 ms and a proximal-to-distal CS activation pattern. Overdrive pacing from the right ventricular apex showed a VAAV response confirming AT as the mechanism. The intracardiac tracings showed regularly irregular narrow complex tachycardia with stable alternating CLs of 410 ms followed by 450 ms **(Figure 4)**. An activation map of the AT was created in the right atrium, which revealed earliest activation on the IAS close to the His deflection **(Figure 5)**. In conjunction with catheter manipulation during mapping, a PFO was identified as the catheter inadvertently entered the left atrium **(Figure 6)**. Sequential mapping of the right-sided IAS revealed the earliest atrial activation at the ostium of the PFO, where a qS complex on the unipolar electrogram was noted. Multiple RFCA lesions created with the CARTO^®^ NAVISTAR^®^ nonirrigated ablation catheter (quadripolar catheter with interelectrode spacing of 1 mm × 7 mm × 4 mm; Biosense Webster, Diamond Bar, CA, USA) were delivered at the peri-PFO, which resulted in successful termination of the AT. Post-RFCA, the tachycardia was noninducible using the programmed electrical stimulation. Since the PFO was missed during the echocardiogram study, it was presumed to be small. The ablation catheter was probably occlusive and the exact site of origination around the PFO (border versus limbus) cannot be commented on.

Postablation, on telemetry, she had no further recurrences of AT and underwent an uneventful CABG procedure. She has been asymptomatic and arrhythmia-free (based on the absence of symptoms) for the past 1.5 years according to periodic outpatient follow-up visits. Repeat TTE showed a left ventricular ejection fraction of 40% to 45% and global hypokinesia of the left ventricle.

## Discussion

Foci for ATs may arise from any location, although up to 63% of cases are located in the right atrial structures, including the tricuspid annulus, crista terminalis, and CS ostium, whereas 37% of them are located in left atrial structures such as the pulmonary veins, mitral annulus, and CS body.^8^ Rarely, FAT may originate from the atrial appendage, IAS, and/or the noncoronary cusp.^5–7,9^ In this report, we described a rare case of FAT arising from the peri-PFO. To the best of our knowledge, this is the first detailed report of FAT originating from the peri-PFO with successful RFCA. Siddiqui et al. briefly reported eight cases of FAT originating from the limbus of a PFO.^10^ Seven of them had an origin of FAT on the left IAS and one patient had an origin on the right IAS of the PFO.^10^ The success rate of FAT RFCA is between 80% and 100%, with an overall complication rate of less than 1% to 2%. The recurrence rates vary between 4% and 27% largely according to the origin of the foci.^1^

Anatomically, PFO is a persistent fetal communication between the atria due to failed flap fusion by the septum primum to the septum secundum within the IAS (posteroinferior portion of the medial atrial wall) region. The prevalence of PFO is approximately 25% to 30% of the population.^5,11^ PFO can be missed during the TTE study, as the technique has a relatively poor sensitivity of 21.4% using color flow Doppler versus 53.8% using bubble contrast for detecting PFO, especially if the patient is obese and the size of the shunt is relatively small.^12^ As such and as was the case in our patient, the PFO was missed during the TTE color flow Doppler study but was later incidentally noted during the EP study and confirmed to be the origin of AT. Perhaps focal mapping with multielectrode diagnostic catheter as described by Price et al.^13^ could potentially better localize the site of origination of the arrhythmogenic focus during the catheter mapping. However, this technique was not used in our patient.

There are three main electrophysiologic mechanisms of AT—namely, enhanced automaticity, triggered activity, and reentry. Although multiple mechanisms for AT with alternating CLs, including automaticity from two separate foci or reentry with variable conduction through an anatomical or functional barrier, have been postulated, the exact mechanism related to the changes in tachycardia CL remains unclear.^14,15^ The tachycardia in our patient had a centrifugal activation pattern from the peri-PFO site. It was an adenosine-sensitive AT. Based on Liu et al.’s^16^ algorithm for the mechanistic diagnosis of AT, the mechanism could possibly have been either triggered activity or enhanced automaticity. Since there was an abrupt onset and termination of AT, the mechanism was likely triggered activity. Our patient demonstrated a regularly irregular AT with alternating CLs of 410 ms and 450 ms, which initially appeared to be a para-Hisian AT. However, this was confirmed to originate from and was treated successfully at the peri-PFO, perhaps suggesting the need for exploration of this site in cases of AT with alternating CLs.

In conclusion, our report describes a rare case of regularly irregular AT with the peri-PFO as the site of origin and demonstrated catheter ablation to be a successful treatment strategy, with the possibility of providing a long-term cure and freedom from antiarrhythmic drugs.
